# Neonatal Tetanus Still Exists: A Case Report and Review of Literature

**DOI:** 10.7759/cureus.61410

**Published:** 2024-05-31

**Authors:** Hanane Hajaj, Hanae Bahari, Anass Ayyad, Sahar Messaoudi, Rim Amrani

**Affiliations:** 1 Department of Neonatology and Neonatal Resuscitation, Mohammed VI University Hospital, Faculty of Medicine and Pharmacy, Mother and Child Health Laboratory, Mohammed I University of Oujda, Oujda, MAR

**Keywords:** opisthotonus, home delivery, spasms, clostridium tetani, neonatal tetanus

## Abstract

Neonatal tetanus (NT) remains the leading cause of death in underdeveloped countries, although it is relatively rare in developed countries. Umbilical stump sepsis in newborns born to unvaccinated mothers is a major risk factor for NT. The World Health Organization describes NT as an infection that affects infants who lose the ability to suck between 3 and 28 days of age, becoming rigid and having spasms. Limited resources in underdeveloped countries have made the management of NT difficult. In this report, we describe a fatal case of NT in a newborn born to a mother who had not received any tetanus toxoid-containing vaccine.

This study aims to make neonatal health professionals aware of the symptoms of NT so that they can diagnose it early and provide the appropriate care to save lives, and stress the importance of tetanus vaccination and maintaining hygienic conditions throughout pregnancy and childbirth to prevent this disease.

## Introduction

Tetanus is a serious and potentially fatal disease caused by *Clostridium tetani*, an anaerobic spore-forming bacterium. When this bacterium enters the body via a breach in a vulnerable host’s skin, it generates a strong neurotoxic compound, also known as tetanospasmin [1]. The main symptoms include muscle rigidity, initially in the face, leading to difficulties with sucking and feeding, followed by rigidity in the neck, trunk, and limbs [2].

Neonatal tetanus (NT) is frequently caused by the use of unsterile instruments to cut the umbilical cord, home birth methods, the application of manure on the umbilical stump, and other unclean traditional practices. In the absence of treatment, the mortality rate is 100% [3]. Autonomic dysfunction is predictive of high mortality [4], especially when NT occurs in neonates less than 7 days old [5]. Furthermore, low birth weight may raise this risk [4]. However, intensive treatment can reduce this rate to 10-20% [3]. Tetanus has an incubation period of 2 to 14 days. Specific symptoms include progressive difficulties in feeding, excessive crying, paralysis or diminished movement, sensitivity to touch, and spasms, with or without opisthotonus. While generalized tetanus is the most prevalent, localized cases can occasionally occur. Trismus is a common presenting sign of generalized tetanus, along with symptoms like risus sardonicus and opisthotonus. Tetanus patients are at risk of asphyxiation and airway obstruction due to spasms in the larynx and respiratory muscles [5].

We present the case of a 6-day-old male neonate with limited prenatal care, born from an unbooked pregnancy with home delivery in unsterile conditions. He was admitted to our intensive care unit for management of NT and received appropriate antispasmodic medications, injectable antibiotics, continuous oxygen therapy, and an injection of anti-tetanus immunoglobulin. Unfortunately, the baby died within 72 hours after admission due to the inability of ventilation.

The goal of this case study is to demonstrate the need for preventive methods such as immunization of pregnant or non-pregnant women with tetanus toxoid and maintaining clean delivery practices with no external application on the cord.

## Case presentation

A full-term male newborn was admitted to our neonatal intensive care unit at the age of 6 days. He was born into a non-consanguineous marriage and an unbooked pregnancy. He presented with tonic spasms, feeding difficulties, a high fever, and excessive crying. The mother, a 40-year-old woman, had not received the tetanus toxoid-containing vaccine. The delivery was assisted by the woman’s mother, who cut the cord using scissors in an unsterile manner and covered the cord as well as the scrotum with a mixture of henna and other traditional plants.

The newborn was not evaluated by a healthcare professional until symptoms appeared. The first 3 days of the newborn’s life were without incident. However, two days before admission, the mother noticed excessive crying, progressive nursing difficulties, and body stiffness, particularly when touched. By the 6th day of life, breastfeeding had become impossible, and a fever had developed.

**Figure 1:**
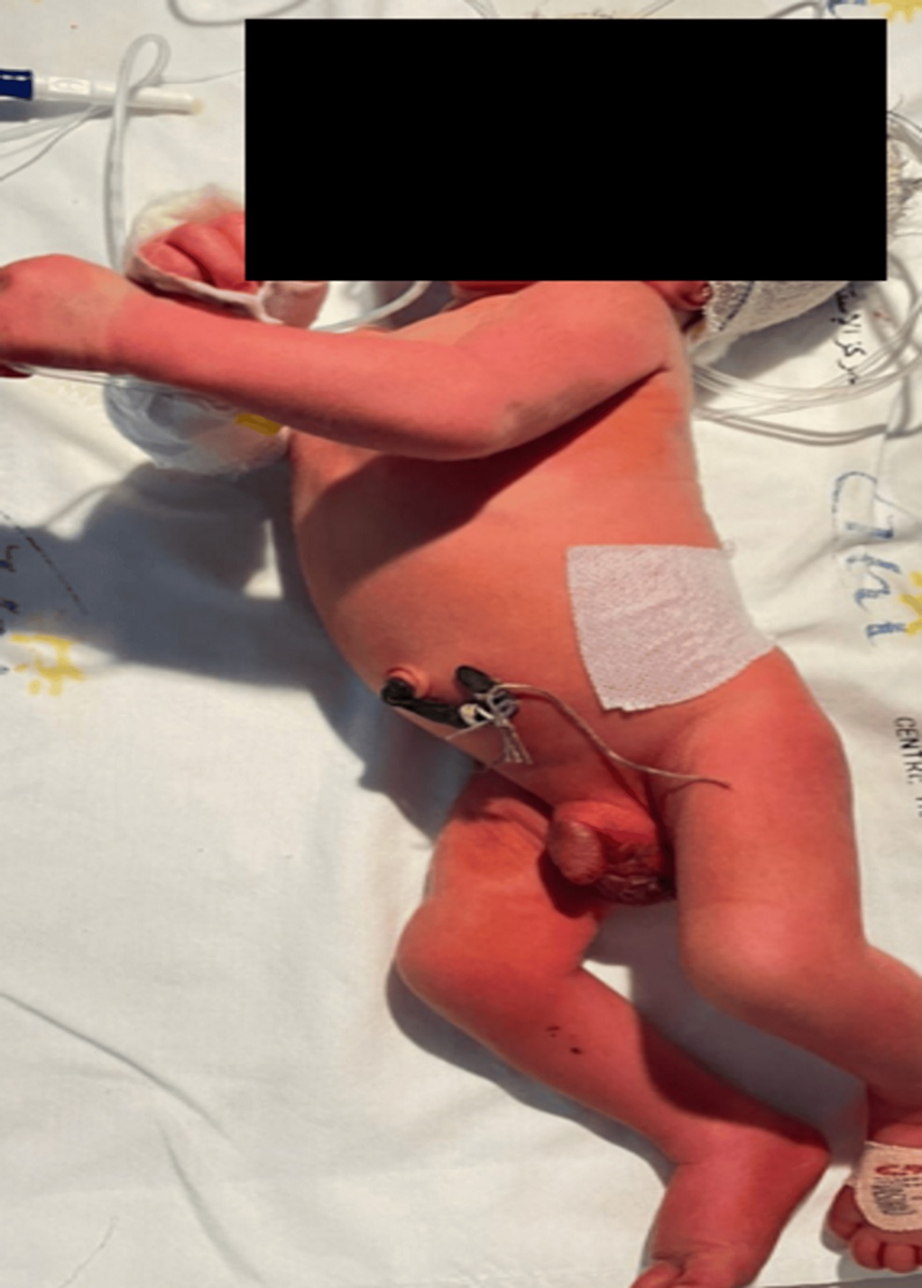
Clinical presentation of our patient including stereotypical hyperextension and posturing with the presence of an unsterile thread attached to the cord.

Upon admission, the physical examination indicated that the newborn weighed 2800 g and had a temperature of 38.5°C. His respiratory rate was 56 breaths per minute, his heart rate was 159 beats per minute, and his oxygen saturation (SpO2) was 98% while receiving 1 L of oxygen per minute. Clinically, the newborn demonstrated generalized hypertonicity with extended limbs. He also had generalized spasms, which were stimulus-sensitive (light and sound) (Figure 1).

**Figure 2:**
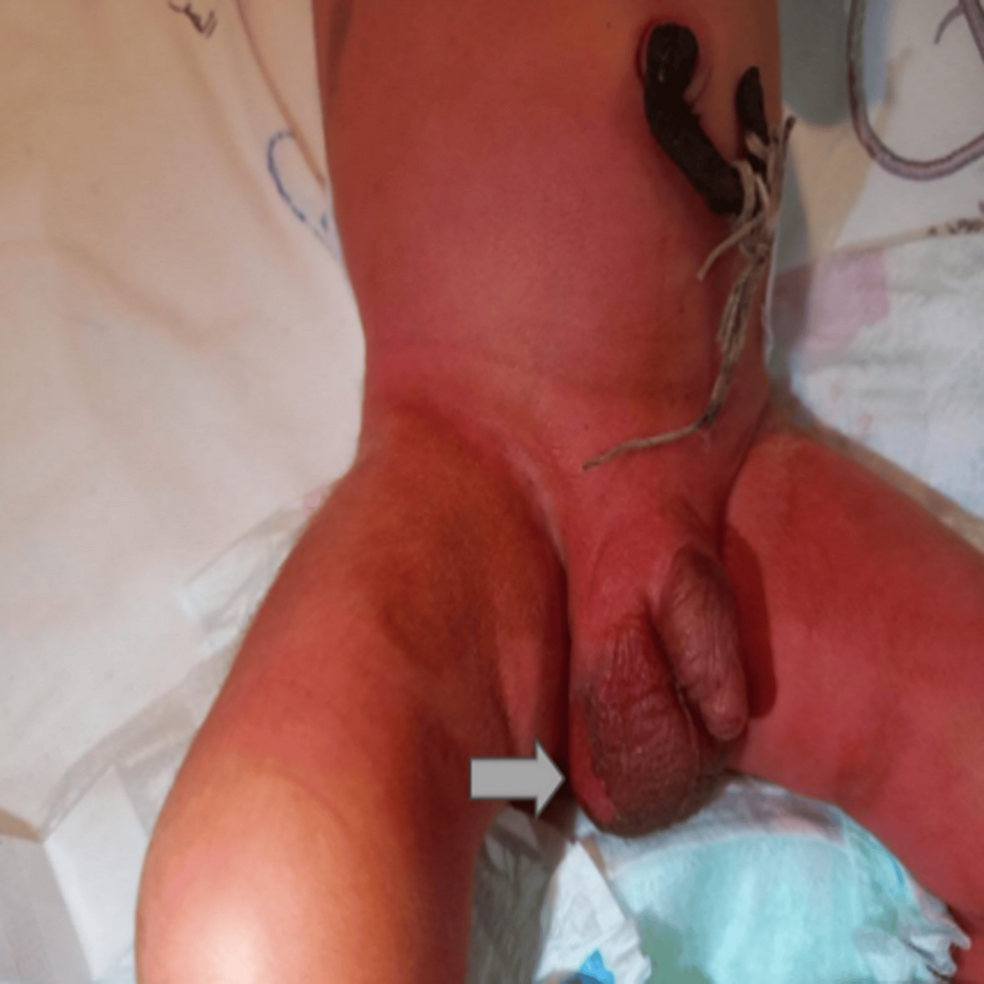
Appearance of our patient’s umbilical cord and ulcerated scrotum.

On abdominal examination, the newborn had a colored umbilicus following the application of a mixture of henna and other traditional plants. Additionally, ulcerative lesions were observed on the right scrotum (Figure 2).

Given this clinical presentation, the diagnosis of NT was made, but various diagnoses were explored, such as drug intoxication, which was ruled out through anamnesis and testing for toxic substances in the urine and blood. Furthermore, the diagnosis of neonatal meningitis was excluded based on the results of paraclinical investigations.

The newborn’s blood count was normal for his age, with a granulocyte count of 12,790 cells/μL and a hemoglobin level of 15.7 g/dL. Biochemical results revealed normal renal function and serum electrolytes (Table 1).

**Table 1:** Biological results of our patient at admission. SGOT: serum glutamic oxaloacetic transaminase; SGPT: serum glutamic pyruvic transaminase

Laboratory Parameter	Values	Reference Ranges
Hemoglobin	15.7 (g/dL)	13.5-19 (g/dL)
White blood cells	12,790 (/µL)	10,000-25,000 (/µL)
Lymphocyte	4500 (/µL)	2000-17,000 (/µL)
Neutrophil	7500 (/µL)	3600-15,500 (/µL)
Platelets	335,000 (/µL)	150,000-400, 000 (/µL)
C-reactive protein	3.6 (mg/L)	0-5 (mg/L)
Creatinine	3.7 (mg/L)	2.4-8.5 (mg/L)
Urea	0.21 (g/L)	0.07-0.33 (g/L)
SGPT	12 (UI/L)	5-34 (UI/L)
SGOT	9 (UI/L)	5-55 (UI/L)
Calcium	94 (mg/L)	88-108 (mg/L)
Potassium	3.5 (mEq/L)	3.4-4.7 (mEq/L)
Sodium	135 (mEq/L)	135-145 (mEq/L)

The blood cultures revealed no bacterial growth, and the cerebrospinal fluid examination revealed clear fluid with appropriate glucose and protein levels, as well as normal Gram staining (Tables 2-3).

**Table 2:** Cerebrospinal fluid (CSF) analysis of our patient.

Biological Sample	
Appearance	Clear
Color	Colorless
Cytology	
White blood cells	0.00/mm³
Red blood cells	0.00/mm³
Microscopic examination following Gram staining	
Microscopic examination following Gram 1 staining	Absence of bacterial flora
Research for soluble antigens	
Research for soluble antigens	Nothing to report
Culture	
Aerobic culture	Negative
Anaerobic culture	Negative
Viral	Negative

**Table 3:** Biochemical analysis of the cerebrospinal fluid (CSF).

Chemical Examination of CSF		
Parameter	Results	Reference ranges
Glucose	3.52 (mmol/L)	3.3-4.4 (mmol/L)
Protein	0.004 (mmol/L)	0.02-0.05 (mmol/L)
Chloride	126 (mmol/L)	110.00-130.00 (mmol/L)
Phosphorus	0.5 (mmol/L)	0.4-0.7 (mmol/L)
Potassium	2.7 (mmol/L)	2.6-3.0 (mmol/L)

A cranial transfontanellar ultrasound was performed, with no abnormalities.

After establishing the diagnosis of NT, the therapeutic approach involved basic care for both the umbilical cord and the ulcerated scrotum, management of spasm and muscle rigidity using an infusion of diazepam and phenobarbitone, intravenous antibiotic therapy with metronidazole, penicillin G, and gentamicin, administration of anti-tetanus immunoglobulin, in addition to continuous oxygen therapy, followed by endotracheal intubation, to provide airway protection.

Unfortunately, the neonate died within 72 hours of admission due to his inability to ventilate.

## Discussion

NT is a potentially fatal bacterial infection caused by *C. tetani* that has been associated with poor hygiene practices during childbirth and the early neonatal period [4], as described in our case report. Maternal and NT (MNT) continues to be a leading cause of neonatal mortality, particularly in low-income countries, where case fatality rates range from 80% to 100% [6]. In 1989, the World Health Assembly supported the elimination of neonatal tetanus (MNTE). This initiative was relaunched in 1999 as the MNTE initiative, targeting 59 priority countries. Worldwide, the percentage of newborns protected from tetanus increased from 74% in 2000 to 86% in 2020. In addition, the percentage of deliveries accompanied by a trained birth attendant climbed from 64% in 2000 to 83% in 2014-2020. The number of reported NT cases globally declined by 88%, from 17,935 in 2000 to 2,229 in 2020. Additionally, estimated deaths were reduced by 92%, decreasing from 170,829 in 2000 to 14,230 in 2019. By December 2020, it was confirmed that 47 (80%) of the 59 priority countries had reached MNTE [7].

Maintaining good care of a newborn’s umbilical cord is extremely important in the initial phase of life, as inadequate practices have been associated with infections [8]. The use of substances on a newborn’s cord stump is strongly associated with a higher risk of omphalitis and sepsis [9-10].

Tetanus is a clinical diagnosis, requiring a high index of suspicion since confirmatory testing is not routinely available. The majority of NT cases (90%) occur between the first 3 and 14 days of life, with symptoms usually appearing between 6 and 8 days [4].

Tetanus symptoms that lead to a clinical diagnosis include the newborn’s inability to suckle at birth and in the first few days following birth, in addition to spasms, stiffness, and convulsions. Trismus, a typical symptom of generalized tetanus, frequently accompanies other features such as spasms of the neck and back muscles (opisthotonus) and facial muscle spasms, which can lead to a distorted grinning expression known as risus sardonicus. It’s possible to confuse these clinical symptoms for seizures. The fundamental distinction is that tetanus preserves consciousness, in contrast to seizures. Clinical symptoms similar to meningitis, hypocalcemia, and hypoglycemia may also occur. Lee and Lin discovered that 70% of tetanus patients experienced symptoms of an umbilical infection, such as discharge, erythema, edema, pain, and localized warmth [11].

NT is routinely treated with tetanus immunoglobulin and wound care. When benzodiazepines alone fail to control spasms in situations with adequate resources, the combination of neuromuscular blockade and ventilation has shown promising outcomes [11]. These treatments manage spasms of respiratory muscles and minimize the risk of death from respiratory muscle failure, which is a leading cause of mortality in NT. Magnesium sulfate causes muscular relaxation, vasodilation, and a decrease in heart rate, which may help to reduce autonomic dysfunction [12]. Other components of case management include eliminating *C. tetani* using broad-spectrum antibiotics, debridement, and disinfecting the umbilical cord with hydrogen peroxide and surgical alcohol [13]. While penicillin has been advised in the past, metronidazole is the current drug of choice and is associated with decreased fatality rates [14]. The optimum strategy for neutralizing circulating tetanus toxin is to administer intramuscular tetanus immunoglobulins (TIG) [13]. The prognosis of newborn tetanus is primarily determined by the incubation and onset periods. Short incubation and onset periods are linked to increased mortality and illness severity [15].

Maintaining tetanus elimination necessitates continual investment in public health, which can be difficult in low-income countries with limited resources for administering the booster immunizations necessary for long-term immunity.

## Conclusions

NT, a rare but deadly disease, frequently affects newborns born at home in areas with limited resources. It is typically lethal without timely intervention; many cases may go unrecognized or fail to reach medical care. Factors contributing to its occurrence include insufficient maternal vaccination, unsterile delivery, and improper umbilical cord care. Immunoglobulin, antibiotics, and supportive interventions are the major therapeutic approaches. Clinicians must be vigilant about this condition.
